# Illuminating the diversity of carotenoids in microalgal eyespots and phototaxis

**DOI:** 10.1080/15592324.2023.2257348

**Published:** 2023-09-19

**Authors:** Shun Tamaki, Tomoko Shinomura, Keiichi Mochida

**Affiliations:** a Bioproductivity Informatics Research Team, RIKEN Center for Sustainable Resource Science, Yokohama, Japan; b Department of Biosciences, Faculty of Science and Engineering, Teikyo University, Tochigi, Japan; c Microalgae Resource Upcycling Research Laboratory, RIKEN Baton Zone Program, Yokohama, Japan; d Kihara Institute for Biological Research, Yokohama City University, Yokohama, Japan; e School of Information and Data Sciences, Nagasaki University, Nagasaki, Japan

**Keywords:** Microalgae, carotenoids, eyespot, phototaxis, diversity, Chlamydomonas, Euglena

## Abstract

Photosynthetic organisms biosynthesize various carotenoids, a group of light-absorbing isoprenoid pigments that have key functions in photosynthesis, photoprotection, and phototaxis. Microalgae, in particular, contain diverse carotenoids and carotenoid biosynthetic pathways as a consequence of the various endosymbiotic events in their evolutionary history. Carotenoids such as astaxanthin, diadinoxanthin, and fucoxanthin are unique to algae. In microalgae, carotenoids are concentrated in the eyespot, a pigmented organelle that is important for phototaxis. A wide range of microalgae, including chlorophytes, euglenophytes, ochrophytes, and haptophytes, have an eyespot. In the chlorophyte *Chlamydomonas reinhardtii*, carotenoid layers in the eyespot reflect light to amplify the photosignal and shield photoreceptors from light, thereby enabling precise phototaxis. Our recent research revealed that, in contrast to the β-carotene-rich eyespot of *C. reinhardtii*, the euglenophyte *Euglena gracilis* relies on zeaxanthin for stable eyespot formation and phototaxis. In this review, we highlight recent advancements in the study of eyespot carotenoids and phototaxis in these microalgae, placing special emphasis on the diversity of carotenoid-dependent visual systems among microalgae.

Carotenoids are light-absorbing yellow, orange, or red pigments that are based on a C40 backbone derived from isoprene. Carotenoids function in photosynthesis, photoprotection, and phototaxis and are biosynthesized across photosynthetic organisms, including algae, land plants, and photosynthetic bacteria.

The central pathway of carotenoid biosynthesis involves a common set of enzymes in these photosynthetic organisms. Various carotenoids have arisen and their compositions have diversified over the course of evolution.^1–3^ In the central pathway of carotenoid biosynthesis, geranylgeranyl diphosphate (GGPP) is converted to phytoene, ζ-carotene, and then to lycopene by phytoene synthase (PSY, also called CrtB), desaturases (phytoene desaturase [PDS] and ζ-carotene desaturase [ZDS]), and isomerases (ζ-carotene isomerase [Z-ISO] and prolycopene isomerase [CrtISO]). Lycopene is cyclized by lycopene β-cyclase (LCY) to form β-carotene, which is transformed into zeaxanthin, violaxanthin, and neoxanthin by various enzymes (two types of β-carotene hydroxylases [BCH and the cytochrome P450 CYP97], zeaxanthin epoxidase [ZEP], and neoxanthin synthase [NSY]). Another cyclized form of lycopene, α-carotene, is hydroxylated to produce lutein. Cyanobacteria and land plants exhibit relatively simple carotenoid compositions, with cyanobacteria primarily containing β-carotene and zeaxanthin as major carotenoids, whereas land plants also accumulate violaxanthin, neoxanthin, and lutein.^1,2^ Notably, microalgae exhibit a remarkable diversity in carotenoid compositions, which is thought to have emerged through multiple endosymbiotic events during their evolution. Although primary endosymbiotic algae such as chlorophytes present carotenoid profiles similar to those of land plants, secondary endosymbiotic algae, such as euglenophytes, ochrophytes, and haptophytes, have evolved unique carotenoids such as diadinoxanthin and diatoxanthin,^1,2^ While diadinoxanthin and diatoxanthin are believed to be converted from neoxanthin, the biosynthetic pathways for these conversions remain elusive.^4^ Decoding and harnessing the genes encoding these elusive enzymes would allow us not only to optimize the composition of carotenoids but also to regulate various cellular functions mediated by such unique compounds.

Carotenoids have three primary functions in photosynthesis: light harvesting, photoprotection, and photon quenching in the xanthophyll cycle. First, carotenoids participate in light harvesting by binding to the photosynthetic light-harvesting complex. They absorb light in the blue to green range of the visible spectrum (450 to 550 nm) and transfer the energy to adjacent chlorophyll molecules, thereby facilitating energy capture during photosynthesis by absorbing photons from wavelengths of light not directly absorbed by chlorophyll.^5^ Second, carotenoids play a role in photoprotection. When chlorophyll is photoexcited, the excitation energy is transferred to an oxygen molecule, producing reactive oxygen species (ROS); these strong oxidizing agents are detrimental to cellular membranes, proteins, and DNA. Carotenoids inhibit the production of ROS by receiving energy from the excited chlorophyll. Carotenoids can also be oxidized by directly receiving energy from ROS, thereby eliminating ROS.^6^ The third function of carotenoids involves their central role in the xanthophyll cycle, a mechanism for mitigating high-light stress. Under excessive high-light conditions, carotenoids in the xanthophyll cycle absorb and dissipate the excess light energy as heat. In chlorophytes, the xanthophyll cycle involves violaxanthin and zeaxanthin. At low light intensity, violaxanthin binds to the light-harvesting complex of photosynthesis and is converted to zeaxanthin, which has a lower light-harvesting efficiency. Under high-light conditions, the reverse reaction occurs.^7^

In flagellated microalgae, carotenoids function not only in photosynthesis but also in phototaxis, which is the movement of a motile organism toward (positive phototaxis) or away from (negative phototaxis) a light source. Phototaxis allows microalgae to optimize their photosynthetic activity by moving toward favorable light environments.^8^ In some flagellated microalgae, carotenoids are concentrated in a pigmented organelle known as the eyespot, which plays a vital role in phototaxis. The eyespot consists of a cluster of carotenoid-rich granules known as eyespot globules (Figure 1). This cellular structure is unique to some microalgae and is not observed in land plants. As exemplified in *C. reinhardtii*, whose eyespot globules act as light reflectors and shields (see the next paragraph) but not as photoreceptors,^9^ the eyespot of flagellated microalgae does not always function as a photoreceptor by itself. Instead, it closely colocalizes with a structured component that functions as a photoreceptor, together forming the photoreceptive organelle known as the eyespot apparatus. The light-responsive molecules that serve as photoreceptors differ among microalgal groups. The photoreceptors for phototaxis in *C. reinhardtii* and *E. gracilis* are channel rhodopsins (ChRs) and photoactivated adenylyl cyclase (PAC), respectively Figure 1.^9,10^ These two types of photoreceptors are distinct: ChRs function as light-responsive calcium ion channels, whereas PAC operates as a light-responsive cAMP-generating enzyme. The eyespot apparatus has been observed in a wide range of flagellated microalgal species and is highly diverse, even in terms of ultrastructure and localization.^11,12^ In chlorophytes, ochrophytes, and some haptophytes, the eyespots are localized within the chloroplast, where the eyespot globules form one or several regularly arranged layers.^9^ Conversely, in euglenophytes, the eyespots are located in the cytosol, where the eyespot globules are irregularly arranged Figure 1.^13^ These examples highlight the diversity in composition and structure of the eyespot apparatus in flagellated microalgae. Despite these findings, the diversity of carotenoids concentrated in eyespot globules and their functions in most microalgae remain largely unexplored. Recent research, however, is providing insights into the roles and the diversity of carotenoids in eyespot globules for phototaxis across different flagellated microalgae groups.

**Figure 1.**
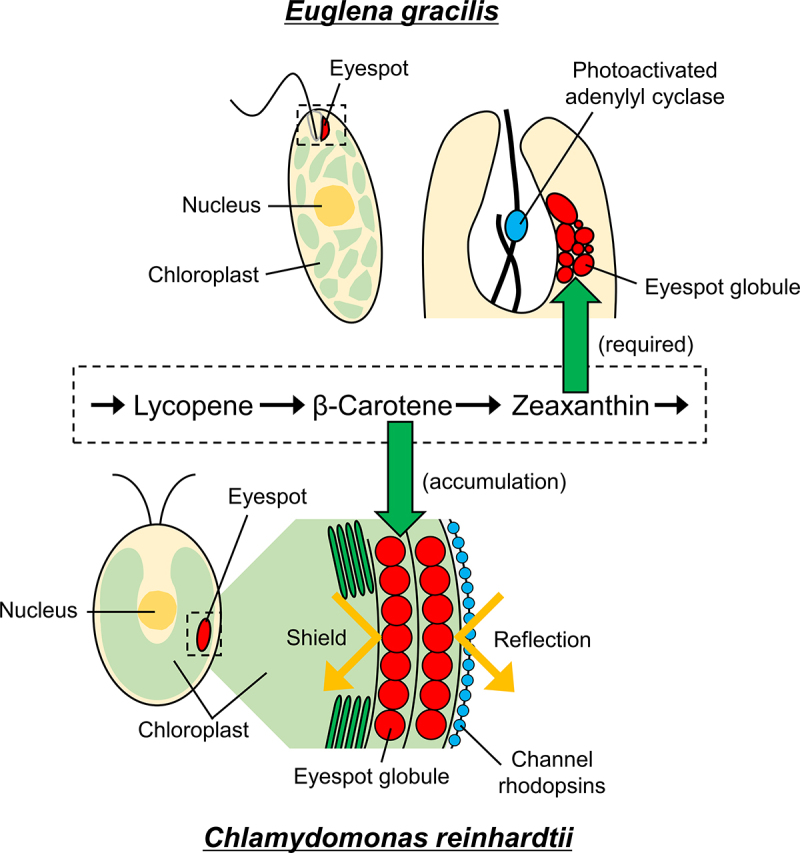
Diagram of eyespot structures, locations, and accumulated carotenoids in *Euglena gracilis* and *Chlamydomonas reinhardtii*. The *E. gracilis* eyespot is located in the cytosol near the photoreceptor photoactivated adenylyl cyclase, where the eyespot globules are irregularly arranged. Zeaxanthin is required for eyespot pigmentation and phototaxis in *E. gracilis*. The mechanistic role of eyespot carotenoids in *E. gracilis* is not yet fully understood. The *C. reinhardtii* eyespot is located in the chloroplast, where the eyespot globules form two regularly arranged layers. These layers of the eyespot reflect light from the outside of the cell, amplifying the light signal received by channel rhodopsins while also shielding light from the inside of the cell. β-carotene is the primary carotenoid in the eyespot of *C. reinhardtii*.

Extensive studies in *C. reinhardtii* have shed light on the mechanisms underlying eyespot-mediated regulation of phototaxis. Because the pigments for the eyespot are carotenoids, it is possible to generate *C. reinhardtii* mutants lacking the eyespot pigment by disrupting genes involved in the carotenoid biosynthetic pathway. However, colorless *C. reinhardtii* strains resulting from mutations in the *PSY* gene are highly light sensitive and unable to grow under light conditions, rendering them unsuitable for phototactic studies.^14^ By contrast,^15^ isolated a green-colored eyespot-less *C. reinhardtii* strain, *lts-211*, that had a point mutation in the *PSY* gene. Although the cellular β-carotene content is markedly lower in the *lts-211* strain, *lts-211* was capable of growth similar to the wild-type strain in the light. *C. reinhardtii* exhibits negative and positive phototaxis under reductive (i.e., photosynthetic) or oxidative (i.e., non-photosynthetic) conditions, respectively.^16^ By contrast, the *lts-211* strain shows a reversed phototactic phenotype when treated with reductive and oxidative reagents, suggesting a misinterpretation of light direction.

The reversed phototaxis behavior of this mutant can be explained by the lens effect of the cell body. In the wild-type strain, the carotenoid layers of the eyespot reflect light from the outside of the cell, amplifying the light signal received by ChRs, while also shielding light from the inside of the cell that could affect phototaxis. By contrast, the *lts-211* strain lacks the ability to shield concentrated light through the lens effect and strongly senses light from the inside of the cell, resulting in a misinterpretation of light direction. This notion suggests that the carotenoid layers in the *C. reinhardtii* eyespot function as both a light reflector and a shield, contributing to accurate phototaxis (Figure 1). β-Carotene is the primary carotenoid in the eyespot of *C. reinhardtii*; the eyespot globules originate from β-carotene-rich plastoglobules Figure 1.^17^ β-Carotene in the eyespot globules shifts from the *trans*-form to the *cis*-form. Because β-carotene hydroxylase does not react with the *cis*-form of β-carotene, a shift to the *cis*-form probably results in β-carotene not being converted to zeaxanthin^9^. The substantial decrease in β-carotene content observed in the *lts-211* strain further supports the notion that the eyespot of *C. reinhardtii* is rich in β-carotene.^15^

Recent research has revealed remarkable differences in the properties of the eyespots in *E. gracilis* and *C. reinhardtii*. To investigate the mechanism of eyespot function in phototaxis of *E. gracilis*, we analyzed a colorless carotenoid-deficient strain (*carotenoid-less 4* [*cl4*]), which lacks pigmented eyespots. This strain was isolated from a cell population via a knockdown of the *crtB* gene, which encodes a phytoene synthase. Notably, unlike colorless mutants of *C. reinhardtii*, *cl4* is capable of growth in the light.^18^ We also determined that *cl4* does not contain pigmented eyespot globules or exhibit phototaxis, but instead displays photophobic behavior. These findings suggest that while the *cl4* strain retains light perception ability, it lacks phototaxis, indicating that carotenoid accumulation in the eyespot may be essential for phototaxis in *E. gracilis*. Specifically, the *cl4* strain did not show changes in swimming direction in response to alterations in light direction, supporting the crucial role of carotenoids in initiating phototaxis.^19^ These observations highlight the distinctive differences in the phototactic functions of carotenoids in the eyespot in chlorophytes vs. euglenophytes.

Because the *cl4* strain of *E. gracilis* lacks carotenoid biosynthesis as well as chloroplast development and photosynthetic ability, the relationship between phototaxis and chloroplast development in *E. gracilis* has been controversial. To address this question, we analyzed orange strains of *E. gracilis* (*cl1* and *cl3*) that accumulate carotenoids in the eyespot but lack chloroplast development. We demonstrated that, unlike the wild type, the *cl1* and *cl3* strains lacked chloroplast structure and did not accumulate chlorophyll. However, the eyespot structure and phototaxis of these strains were similar to those of the wild type. These results indicate that *E. gracilis* accumulates carotenoids in the cytosolic eyespot globules independently from chloroplast development.^20^ By contrast, *C. reinhardtii* synthesizes carotenoids and forms the eyespot within the chloroplast. Furthermore, whereas *C. reinhardtii* eyespot carotenoids are primarily β-carotene,^17^ the *cl1* and *cl3* strains of *E. gracilis* mainly accumulate zeaxanthin. In addition, the *E. gracilis* eyespot has been reported to contain carotenoids such as β-carotene, diadinoxanthin, and diatoxanthin.^21^

To further explore the relationship between eyespot formation and carotenoid composition in *E. gracilis*, we used clustered regularly interspaced short palindromic repeat (CRISPR)/CRISPR-associated nuclease 9 (Cas9)-mediated genome editing to generate a collection of *E. gracilis* mutants in which each of 16 carotenoid biosynthetic genes was independently targeted. Among these mutants, the *lcy* mutants and *cyp97h1 cyp97f2* double mutants, which lack zeaxanthin, failed to form the red eyespot and did not exhibit phototaxis. By contrast, the *cyp97h1* single mutants, which contained zeaxanthin, formed a pigmented eyespot and exhibited phototaxis similar to the wild type. These findings indicate that zeaxanthin is required for phototactic eyespot formation in *E. gracilis*.^22^ Collectively, these observations suggest that euglenophytes and chlorophytes have evolved chemically and physiologically distinct eyespots (Figure 1). However, eyespot formation and the mechanistic role of eyespot carotenoids in *E. gracilis* are still poorly understood, necessitating further investigation to identify the underlying mechanisms of eyespot formation and phototaxis. A comprehensive understanding of the eyespot-mediated visual system in microalgae will provide valuable insights into the functionality and utilization of carotenoids.
